# The Promoting Activity in Cancer Survivors (PACES) trial: a multiphase optimization of strategy approach to increasing physical activity in breast cancer survivors

**DOI:** 10.1186/s12885-018-4662-5

**Published:** 2018-07-18

**Authors:** Chad D. Rethorst, Heidi A. Hamann, Thomas J. Carmody, Kendall J. Sharp, Keith E. Argenbright, Barbara B. Haley, Celette Sugg Skinner, Madhukar H. Trivedi

**Affiliations:** 1 University of Texas Southwestern Medical Center, 5323 Harry Hines Blvd, Dallas, TX 75390 USA; 2 University of Arizona, 1503 E University Blvd, Tucson, AZ 85721 USA

## Abstract

**Background:**

Despite the significant, empirically supported benefits of physical activity, the majority of breast cancer survivors do not meet recommended guidelines for physical activity. A variety of effective strategies to increase physical activity in breast cancer survivors have been identified. However, it is unknown which of these strategies is most effective or how these strategies might be combined to optimize intervention effectiveness.

**Methods:**

The proposed trial uses multiphase optimization strategy (MOST) to evaluate four evidence-based intervention strategies for increasing physical activity in breast cancer survivors. We will enroll 500 breast cancer survivors, age 18 and older, who are 3-months to 5 years post-treatment. Using a full-factorial design, participants will be randomized to receive a combination: 1) supervised exercise, 2) facility access, 3) self-monitoring, and 4) group-based active living counseling. The primary outcome, moderate-to-vigorous physical activity (MVPA) will be measured at baseline, 3 months, and 6 months using an Actigraph GT3X+. To evaluate intervention effects, a linear mixed-effects model will be conducted with MVPA as the outcome and with time (3 months and 6 months) as the within-subjects factor and intervention (i.e., supervised exercise, facility access, self-monitoring, and active living counseling) as the between subjects factor, along with all two-way interactions.

**Discussion:**

The purpose of the PACES study is to evaluate multiple strategies for increasing physical activity in breast cancer survivors. Results of this study will provide in an optimized intervention for increasing physical activity in breast cancer survivors.

**Trial registration:**

Clinicaltrials.gov Identifier: NCT03060941. Registered February 23, 2017.

## Background

For the over 3 million breast cancer survivors in the United States (including at least 135,000 Texans), the post-treatment survivorship period is often accompanied by significant physical and psychosocial health burdens. Five-year recurrence rates for breast cancer survivors range from 7 to 13% [1] with a subset experiencing an increased risk for other cancers [2]. Breast cancer survivors also have significant medical comorbidities, symptom burdens, and late effects that decrease quality of life and affect prognosis [3]. For example, up to 30% of breast cancer survivors report poor quality of life up to 5 years post-treatment [4, 5]. Almost 40% of breast cancer survivors experience significant psychosocial distress including fatigue, depression, and/or anxiety [6, 7]. Given the numerous health-related challenges faced by breast cancer survivors, it is imperative to provide this population with evidence-based services to improve their physical and mental well-being.

Physical activity has consistently been shown to significantly improve disease outcomes and reduce mortality for breast cancer survivors, along with improvements in physical and psychosocial health. Multiple epidemiologic studies have shown that adequate physical activity is associated with decreased risk of disease recurrence, breast cancer-specific mortality as well as all-cause mortality, along with better quality of life and improved physical functioning [8–11]. Results from a prominent meta-analysis reported that post-diagnosis physical activity reduced breast cancer-specific mortality by 24%, all cause mortality by 41% and disease recurrence by 24% [12]. Another meta-analysis links post-treatment physical activity with improved cardiorespiratory fitness, increased upper/lower body strength, reduced fatigue, improved quality of life, reduced anxiety and increased self-esteem [13].

Based on the overwhelming evidence supporting beneficial effects of physical activity in breast cancer survivors, the American Cancer Society (ACS), the National Comprehensive Cancer Network (NCCN), and the American College of Sports Medicine (ACSM) have all adopted recommendations for physical activity among cancer survivors and promoted evidence-based interventions to increase physical activity in cancer survivors. Organizational consensus is that cancer survivors get a *minimum of 75 min vigorous or 150 min moderate activity per week*. In 2010 the ACSM published a comprehensive set of guidelines for physical activity among cancer survivors, concluding that exercise is safe and effective for breast cancer survivors and results in many physical and psychosocial improvements [14]. Information from these guidelines provides an excellent blueprint for assessing physical activity readiness and understanding evidence-based outcomes.

Despite the significant, empirically supported benefits of physical activity, the majority of breast cancer survivors do not meet recommended guidelines for physical activity and have great need for feasible and evidence-based interventions. National epidemiologic studies [8, 10, 15, 16] indicate that approximately two-thirds of breast cancer survivors do not meet physical activity recommendations, including at least one-third of patients who engage in *no* regular physical activity. Numerous interventional strategies have been identified as efficacious for increasing physical activity among cancer survivors, ranging from brief physical activity screening, education, and self-monitoring to more intensive lifestyle counseling and on-site provision of exercise equipment and monitoring of activity levels. However, many of these resource-intensive interventions are not available for the majority of breast cancer survivors, and are often not feasible even if available. Such services are rarely covered under insurance plans or offered within standard oncologic care [17]. Even less formal strategies for promoting physical activity are underutilized. For example, surveys of oncologists indicate that approximately 50% do not routinely advise patients to engage in physical activity [18, 19]. This lack of practical physical activity services is in contradiction to the vast evidence not only demonstrating the benefit of physical activity, but also the evidence supporting several behavioral strategies for increasing physical activity among breast cancer survivors. In addition, it is unclear how these strategies might be combined to maximize outcomes in clinical settings. Therefore, there is a crucial need to provide feasible evidence-based physical activity interventions to cancer survivors and understand the most efficacious components of these interventions.

## Study objectives

Although multiple strategies for increasing physical activity have proven efficacious, little is known about the optimal intervention strategies for breast cancer survivors or how those interventions can be effectively implemented in real-world settings. This project will assess the implementation of evidence-based strategies for increasing physical activity among breast cancer survivors. The study utilizes multi-phase optimization strategy (MOST) to identify the optimal combination of intervention strategies to increase physical activity among breast cancer survivors.

### Aim 1

Provide education and evidence-based interventions to increase physical activity among breast cancer survivors treated at the Simmons Cancer Center and Parkland Health and Hospital System.

#### Aim 1A

Provide evidence-based education about physical activity to 4500 breast cancer survivors.

#### Aim 1B

Deliver intensive evidence-based physical activity interventions to 500 survivors who are not meeting physical activity recommendations.

### Aim 2

Evaluate changes in physical activity and identify the optimal intervention or combination of interventions for increasing physical activity in breast cancer survivors who are not meeting physical activity guideline recommendations at baseline.

#### Aim 2A

Measure physical activity at baseline and follow-up periods (3- and 6-months post-baseline) and assess percentage of survivors meeting physical activity guideline recommendations.

#### Aim 2B

Using the Multiphase Optimization Strategy framework, compare improvements in physical activity across intervention components utilized for breast cancer survivors.

#### Aim 2C

Evaluate secondary outcomes including health-related quality of life and psychosocial functioning.

#### Aim 2D

Evaluate psychosocial factors as predictors of physical activity behavior change.

### Aim 3

Evaluate program acceptability and program satisfaction outcomes to assess potential for dissemination and implementation of the PACES program.

## Study design

All study procedures described below have been approved by the UT Southwestern Institutional Review Board (IRB). Any change to the study protocol will be submitted to the UT Southwestern IRB for approval prior to implementation. Through individually based, mail and in-person recruitment, we will provide physical activity education to 4500 female breast cancer survivors who were treated at the outpatient oncology clinics associated with the UT Southwestern Harold C. Simmons Comprehensive Cancer Center, including those treated at Parkland Health and Hospital System. All eligible breast cancer survivors (defined as being between 3 months and 5 years post-treatment) will be contacted through either the outpatient clinic setting (during post-treatment appointments) or by mail (with contact information from the cancer registries associated with the outpatient settings of the Simmons Cancer Center and Parkland Health and Hospital System). Through this initial contact, eligible breast cancer survivors will receive: 1) evidence-based educational materials about physical activity recommendations for cancer survivors, 2) a brief questionnaire about their current physical activity level, 3) an invitation to participate in a physical activity program.

Survivors who indicate interest will be contacted by the project team and scheduled for a baseline visit. At this session, informed consent will be obtained and participants will complete further baseline assessments about physical activity and other psychosocial and behavioral indicators. Following a 7-day physical activity assessment, we will randomize participants into evidence-based intervention component groups, including self-monitoring, active living classes, supervised exercise sessions, and facility access memberships, and compare physical activity outcomes (assessed 3- and 6-months post-baseline) between groups. This process will allow us to understand which intervention components are most effective for breast cancer survivors. Findings from this project will inform future physical activity programs by pinpointing the most effective components of intervention for breast cancer survivors. Furthermore, we will assess factors that influence dissemination and implementation of the PACES program. Through this process, we will be able to further refine the program to ensure it can be implemented across the state of Texas.

## Study population

All breast cancer survivors between 3 months and 5 years post-treatment will be contacted via postal mail to participate in a brief online survey. Follow-up emails will be sent to all survivors with available email addresses. We will determine initial eligibility based on survey responses. Inclusion/exclusion criteria for PACES are as follows.

### Inclusion criteria

1. breast cancer survivors between 3 months and 5 years post-treatment (chemotherapy, radiation, or surgery)
2. report < 150 min of weekly moderate-to-vigorous physical activity (MVPA) on the IPAQ
3. physically able to engage in physical activity

### Exclusion criteria

1. medical condition contraindicating physical activity participation
2. cognitively unable to give informed consent

## Subject recruitment/screening

Through individually based, mail and in-person recruitment, we will provide physical activity education to 4500 female breast cancer survivors who were treated at the outpatient oncology clinics. All eligible breast cancer survivors (defined as being between 3 months and 5 years post-treatment) will be contacted through either the outpatient clinic setting (during post-treatment appointments) or by mail (with contact information from the settings’ associated cancer registries). Through this initial contact, eligible breast cancer survivors will receive: 1) evidence-based educational materials about physical activity recommendations for cancer survivors, 2) a brief questionnaire about their current physical activity level, 3) a link to the program website that will include further information on the benefits of physical activity and advice for being more active, 4) an invitation to participate in a physical activity program. Strategies for recruitment and retention will be assessed in focus groups, which will be conducted separately by clinic (see below). We will implement different strategies if they are deemed necessary through these focus groups.

## Screening

Survivors will be asked to complete the brief online screening questionnaire that includes the a link to a brief questionnaire about current physical activity behaviors (International Physical Activity Questionnaire [IPAQ]) and safety of engaging in physical activity (Physical Activity Readiness Questionnaire [PAR-Q]). The screening questionnaire may also be completed via phone if survivors do not have access to a computer. Survivors who complete the questionnaire (regardless of their preference for future contact) will be randomly selected to receive a Fitbit as compensation for their time and effort.

**Fig. 1:**
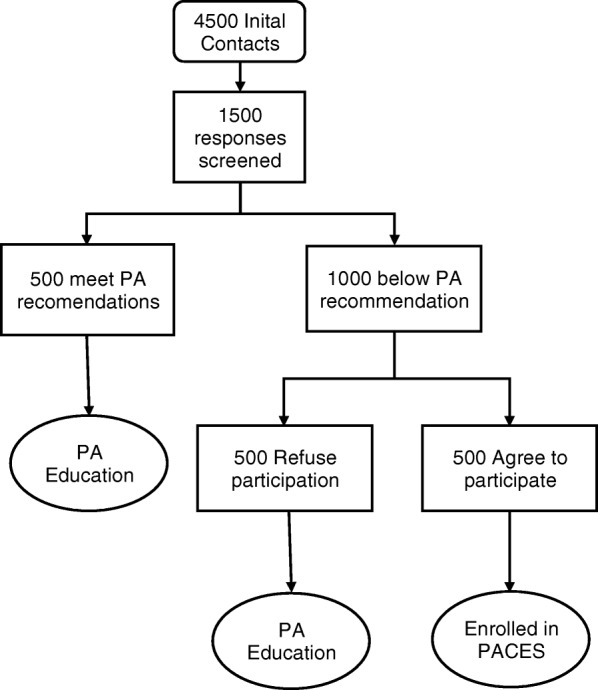
Estimated participant recruitment

Based on previously published work utilizing contact of breast cancer survivors from the cancer registry system [20], we anticipate that approximately one-third (33%) of the contacted survivors (*n* = 1500) will return the questionnaire and agree to future contact to learn more about further physical activity interventions. Based on indications of physical activity frequency from the online questionnaire, we will contact those who do not currently meet physical activity recommendations and invite them to attend an initial in-person session. This focus on sedentary cancer survivors maximizes use of resources and focuses on those in greatest need of intervention. Individuals who indicate interest in further contact and are currently meeting or exceeding ACSM physical activity guidelines will be provided with educational materials via the study website and given project contact information if they have further questions, but will not be invited for an initial in-person evaluation session (see Fig. 1).

## Enrollment and randomization

Potential participants identified through the screening process will be contacted by phone and invited for a baseline assessment. At this baseline visit, study personnel will explain details of the study to potential participants and give them time to read through the informed consent document. Study personnel will then go through the document and answer any questions. Potential participants who choose to provide informed consent and sign the informed consent form will then proceed to the baseline assessment.

Interested cancer survivors will meet with a trained project interventionist and be assessed for safety of engaging in physical activity, following recommendations identified in the ACSM guidelines (described in Section “Study Design” [14];). The Physical Activity Readiness Questionnaire (PAR-Q) [21] is a self-report questionnaire designed to assess safety of engaging in physical activity. Survivors indicating contraindications to physical activity on the PAR-Q will be asked to meet with a project physician to determine safety prior to project enrollment. In addition, lymphedema and pain will be assessed at baseline and throughout the program to ensure participant safety.

After study eligibility has been confirmed, survivors will complete an baseline assessments, which will involve of collection of demographic data, self-report and objective measurement of physical activity, and psychosocial predictors of physical activity behavior change (see Section 6.2.3 for full description of outcome assessments). Baseline physical activity will be measured objectively with an Actigraph GT3X+ accelerometer. Survivors will be asked to wear the Actigraph for a 7-day period and will be scheduled for a randomization visit to be held at the conclusion of this 7-day period.

At the randomization visit, participants will return their Actigraphs and be randomized into an intervention group. Randomization will be stratified by clinical site (UT Southwestern vs. Parkland). The randomization scheme, conducted by the study statistician (Dr. Carmody), will consist of balanced blocks within each stratum with block sizes varied and randomly permuted. Randomization tables will be uploaded in RedCap and allocation will occur following completion of baseline assessments.

## Study interventions

The 500 participants in the physical activity intervention will be randomly assigned to one of sixteen intervention groups (see Table 1).

**Table 1:** Randomization schema

Intervention	Group 1	Group 2	Group 3	Group 4	Group 5	Group 6	Group 7	Group 8	Group 9	Group 10	Group 11	Group 12	Group 13	Group 14	Group 15	Group 16
PA education	x	x	x	x	x	x	x	x	x	x	x	x	x	x	x	x
Facility Access		x				x	x	x				x	x	x		x
Supervised Sessions			x			x			x	x		x	x		x	x
Self-monitoring				x			x		x		x	x		x	x	x
Active Living Class					x			x		x	x		x	x	x	x

### Physical activity enhanced education (“PA education”)

All groups will receive enhanced education focused on physical activity. Provision of print-based materials can result in significant increases in physical activity. All groups in the study will be given a copy of *Exercise for Health: An Exercise Guide for Breast Cancer Survivors* [22, 23], a 56-page book based on the theory of planned behavior. The book was developed and evaluated by experts in the field [23] and has been proven efficacious in increasing physical activity in breast cancer survivors, as demonstrated in a study by Vallance et al. [22] in which breast cancer survivors who received the book increased their physical activity levels by 70 min per week [22]. Topics covered within the book include benefits of exercise in breast cancer survivors; recommendations on type, duration, frequency and intensity of exercise; goal-setting; and advice on overcoming common barriers.

### Supervised exercise sessions

Results of a recent study suggest that a physical activity program that includes supervised exercise sessions may be more effective in increasing sustained physical activity [24]. Participants randomized to this intervention will attend supervised exercise sessions. The participant’s weekly exercise goal will be 150 min, which will be completed via the supervised exercise sessions and home sessions. In Weeks 1–2, participants will complete 3 supervised exercise sessions with a trained exercise interventionist in the Exercise Lab located at UT Southwestern. In Weeks 3–4, participants will complete 2 supervised sessions along with at least 2 home exercise sessions. In Weeks 5–6, participants will complete 1 supervised session along with at least 3 home exercise sessions.

### Facility access

Access to exercise facilities has been associated with increased engagement in physical activity [25, 26] and access to exercise facilities are often utilized in effective physical activity promotion interventions [27]. Participants randomized to this intervention will receive a 6-month membership to a local fitness facility. We have arranged for memberships at the at a variety of city-operated and private fitness facilities to ensure convenient access for all participants.

### Self-monitoring

Substantial evidence supports self-monitoring techniques to increase physical activity [28, 29]. In a large study of breast cancer survivors, the use of pedometers resulted in significant physical activity increases in individuals who were previously sedentary [22]. Technological advances have resulted in devices utilizing tri-axel accelerometers that provide more accurate physical activity devices. Furthermore, these devices can be synced to smartphones and computers for more automated self-monitoring. Participants randomized to this intervention will be provided with a commercially available activity monitor (Fitbit Alta HR). The project interventionist will instruct subjects on proper use of the device and options for viewing the data collected by the device. Subjects will be instructed to wear the device daily. These devices are compatible with both Android and Apple phones or can be synced with any computer with internet access. Individuals without access to a compatible device will be given paper logs and instructed to record their activity counts provided by the activity monitor on a daily basis.

### Active living counseling

Lifestyle interventions are capable of producing significant, long-lasting increases in physical activity [30]. We will utilize a lifestyle counseling intervention based on the Active Living Every Day program (ALED). ALED is grounded in the Transtheoretical Model and Social Cognitive Theory and has been demonstrated as an effective intervention for increasing physical activity across several populations [30–32]. The lifestyle counseling program will consists of 12 in-person group educational sessions, facilitated by project interventionists. Participants randomized to this intervention will attend 12 bi-weekly sessions. Interventionists will be trained in delivery of the ALED program. These sessions will involve discussion of topics related to increasing physical activity, including: identifying and overcoming barriers, setting goals, social support, and time management.

## Outcome assessments

Study assessments will be conducted at baseline, 3 months, and 6 months. Participants will be reimbursed for completion of study assessments. Patient self-report data will be directly entered in to the RedCap data management system.

### Measurements of physical activity

#### Actigraph GT3X+ accelerometer

The Actigraph will provide a valid and reliable objective assessment of physical activity [33]. Subjects will be asked to wear the device for a 7-day period at each assessment time point (baseline and weeks 13 and 25). Subjects will be instructed to wear the device on their waist and to remove the device only when it may become submerged in water (bathing, swimming, etc.). Following, the 7-day period, subjects will return the accelerometers using a postage-paid envelope.

#### International Physical Activity Questionnaire – Short form (IPAQ)

The IPAQ [19] is a 7-item scale designed to assess physical activity.

#### Exercise Vital Sign (EVS)

The EVS [34] is a 2-item scale that is used to estimate an individual’s physical activity.

### Measurement of psychosocial and physical outcomes

#### Quick Inventory of Depressive Symptomatology – Self-Rated (QIDS-SR_16_)

The QIDS-SR_16_ [35–37] is a 16-item questionnaire to assess severity of depression-specific symptoms. The QIDS-SR_16_ has high reliability (Cronbach’s alpha of 0.83), good concurrent validity (correlations between the QIDS-SR_16_ and the 17-item Hamilton Rating Scale for Depression is 0.81) [35].

#### Pittsburgh Sleep Quality Index (PSQI)

The PSQI [38] is a 19-item scale designed to assess sleep quality and disturbances. Scores range from 0 to 21 with higher scores representing worse sleep quality. The PSQI has demonstrated acceptable reliability (Cronbach’s alpha of 0.80) in the assessment of self-reported sleep quality and validity when compared to sleep diaries and polysomnography [39].

#### Brief Fatigue Inventory (BFI)

The BFI [40] is a 9-item scale designed to assess fatigue. The BFI demonstrates good reliability (Cronbach’s alpha of 0.96) and validity (correlation of 0.86 with the POMS fatigue subscale).

#### Functional assessment of Cancer therapy – Breast (FACT-B)

The FACT-B [41] is a 44-item scale designed to measure multidimensional quality of life in breast cancer patients. The FACT-B has demonstrated acceptable reliability (Cronbach’s alpha of 0.90) and concurrent validity (0.87 correlation with the Functional Living Index-Cancer and 0.86 correlation with the Functional Assessment of Cancer Therapy-General).

#### Brief COPE

The Brief Cope [42] is a 28-item scale designed to assess a wide range of coping responses. The Brief COPE has been used in studies of cancer patients and demonstrates good reliability and validity.

#### Pain – Frequency, Intensity, and Burden Scale (P-FIBS)

The P-FIBS [43] is a 4-item scale that assesses the frequency, intensity, and burden of pain.

#### Dimensional Anhedonia Rating Scale (DARS)

The DARS [44] is a 21-item scale that assesses anhedonia.

#### Anthropomorphic assessments

Height will be assessed at baseline. Weight and waist circumference will be measured at each assessment time point. Height and weight measurements will be used to calculate Body Mass Index (BMI).

#### Metabolic indices

Blood samples will be collected by a trained phlebotomist to allow for evaluation of markers of metabolic health, including blood glucose, triglycerides, and lipids.

#### Relationship status

A single-item question will ask participants to indicate their current relationship status from one of 7 categories (single, never married; cohabiting with partner; married, living together; married, not living together; separated; divorced; widowed). The Couples Satisfaction Index (CSI) [45] is a 16-item form that captures relationship quality among participants who endorsed currently being in a romantic relationship. The CSI is a well-validated and reliable measure that draws from other previously established relationship satisfaction measures.

#### Physical activity stages of change questionnaire

The 4-item scale assesses the current stage within the Transtheoretical Model framework (Pre-contemplation, Contemplation, Preparation, Action, Maintenance). These stages are highly correlated with change in physical activity over time [46].

#### Physical activity self-efficacy questionnaire

The 5-item scale assesses self-efficacy for physical activity. Activity-specific self-efficacy is highly correlated with activity change and psychosocial outcomes [46].

## Statistical analysis

The goal of identifying the most effective components of physical activity intervention will be accomplished through the MOST design in which the four interventions (supervised exercise, facility access, self-monitoring, and active living counseling) each at two levels (presence, absence) are included for a total of 16 combinations (see Table 1). The 500 participants will be divided into one of the 16 combinations for about 31 participants per combination. Note that some participants receive more than one intervention so that half the sample (*n* = 250) receives each intervention. All participants completing baseline and at least one post-baseline assessment will be used in the analysis.

To accomplish the analytical goals, a linear mixed-effects model (using SAS Proc Mixed), will be conducted with MVPA as the outcome and with time (3 months and 6 months) as the within-subjects factor and intervention (i.e., supervised exercise, facility access, self-monitoring, and active living counseling) as the between subjects factor, along with all two-way interactions. Baseline minutes of MVPA, age, BMI, race/ethnicity, socioeconomic status, and education will be included as covariates, along with other relevant demographic and clinical characteristics (e.g., disease stage, treatment, time since diagnosis) collected through participant self-report and data available through the UT Southwestern Cancer Registry. The model will allow for random intercepts while all other factors will be fixed effects.

If MVPA is not sufficiently normally distributed, a log transformation will be used. If the log transformation does not sufficiently normalize the data then non-parametric methods or non-linear models will be considered. As recommended in Collins, et al [47–49], effect coding (− 1, 1) of the interventions will be used instead of dummy coding (0, 1). Main effects and interactions will be estimated for the 3 month and 6 month assessments. Effects will be considered significant if p < = 0.05. The goodness of fit of the final model will be assessed.

This model allows an evaluation of overall pre- post-results for participants, along with more sophisticated analyses of which interventional components are most effective. The results of the analysis will be used to determine which of the intervention components should be included in an intervention package to improve physical activity in breast cancer survivors. However, determining which interventions to include in the intervention package is not straightforward in the presence of large interaction effects. Therefore, interventions will be selected according to the hierarchical ordering principle and modified heredity principle as described in Collins, et al [47–49]. Similar analyses will be conducted to examine changes in secondary outcomes including: quality of life (FACT-B), fatigue (BFI), sleep quality (PSQI), and depressive symptoms (QIDS-SR).

To assess psychosocial factors as predictors and moderators of physical activity behavior change, the linear mixed model repeated measures analysis described above for MVPA will be repeated with each psychosocial factor added as a baseline covariate, in an interaction with intervention group, and in a three-way interaction with intervention group and time. A significant baseline covariate effect in the absence of interaction effects will identify the factor as a predictor of behavior change irrespective of intervention while a significant interaction effect will identify the psychosocial factor as a moderator of behavior change. In other words, the effect of the psychosocial factor varies depending on the intervention. Effects will be considered significant if p < = 0.05.

Analyses related to Goal 3 focus on assessment of factors that will influence future dissemination and implementation of the PACES program. We have selected two important outcomes: 1) program acceptability, and 2) program satisfaction outcomes. These outcomes will be analyzed using the same model described above for MVPA.

The single-site approach used for the analyses despite the accrual from the Cancer Center’s two locations (UT Southwestern Center and Parkland) reflects the fact that the intervention will only be delivered at one site, as survivors from both UT Southwestern and Parkland clinics will complete all study procedures at UT Southwestern Medical Center. Differences in patient populations of the two clinics and within each clinic will be accounted for by including race/ethnicity, socioeconomic status, and education as covariates in the analysis. As described above, we will also utilize “clinic” as a stratification factor in the randomization scheme.

### Power analysis

We assume a sample of 500, (one cluster based on single-site setting), testing 4 interventions in a full factorial design (16 groups), and testing of main effects and 2-way interactions. Also, we assume a baseline measure of the outcome to be used as a covariate and a correlation between pre- and post-measures of 0.65 (as assumed in Dziak, Nahum-Shani, and Collins [50]). Given a sample size of 500, a main effect of size 0.191 can be detected with 80% power and an interaction effect of 0.382 can be detected with 80% power. Thus, with a sample size of 500 we can detect small main effects (less than 0.2) and interaction effects that are between small and moderate (0.2 to 0.5) [51].

## Data management

Project data will be entered into an established database developed through RedCap database management software. This software is support by NIH as well as by the Biostatistics Division at UT Southwestern. Relational databases are constructed using a set of two-dimensional tables. Data can be output to standard formats such as Excel, SAS, SPSS. The database will be stored on a secure UT Southwestern network server (backed up nightly) with access limited to project staff. Appropriate procedures safeguard of participant privacy, including data de-identification and SSL encryption for data transfer, will be observed.

## Safety monitoring

Study principal investigators (Drs. Rethorst and Trivedi) will meet monthly to examine accumulating data to assure protection of participants’ safety while the study’s scientific goals are being met. These reviews will include an assessment of the possible relatedness of the event to the study intervention or other study procedures. All study staff will be trained in proper event reporting. All protocol deviations and adverse events will be recorded in RedCap. Unexpected serious adverse events potentially related to study procedures will be reported to the UT Southwestern IRB within 5 working days, as will protocol deviations that increase participant risk or compromise data quality.

## Discussion

The purpose of the PACES study is to evaluate multiple strategies for increasing physical activity in breast cancer survivors. Previous studies have identified numerous strategies that can be effective in promoting physical activity among breast cancer survivors. What remains unknown is which strategies are most effective and how these different strategies may interact with each other. Through rigorous evaluation of program outcomes utilizing Multiphase Optimization Strategy, we will be able to identify optimal combinations of intervention components to increase physical activity among breast cancer survivors.

Results of this study will be reported in a peer-review journal. These results will provide in an optimized intervention for increasing physical activity in breast cancer survivors. However, challenges will remain in implementing the optimized intervention for routine use in diverse clinical settings. Future research will be necessary to evaluate strategies for dissemination and implementation to ensure intervention effectiveness.

## Abbreviations

ACS: American Cancer Society
ACSM: American College of Sports Medicine
ALED: Active Living Every Day program
BFI: Brief Fatigue Inventory
BMI: Body Mass Index
CSI: Couples Satisfaction Index
DARS: Dimensional Anhedonia Rating Scale
EVS: Exercise Vital Sign
FACT-B: Functional Assessment of Cancer Therapy – Breast
IPAQ: International Physical Activity Questionnaire
IRB: Institutional Review Board
MOST: Multiphase optimization strategy
MVPA: Moderate-to-vigorous physical activity
NCCN: National Comprehensive Cancer Network
PACES: Promoting Activity in Cancer Survivors
PAR-Q: Physical Activity Readiness Questionnaire
P-FIBS: Pain – Frequency, Intensity, and Burden Scale
PSQI: Pittsburgh Sleep Quality Index
QIDS-SR: Quick Inventory of Depressive Symptomatology – Self-Rated
